# A peptide corresponding to the neuropilin-1-binding site on VEGF_165_ induces apoptosis of neuropilin-1-expressing breast tumour cells

**DOI:** 10.1038/sj.bjc.6602308

**Published:** 2005-01-18

**Authors:** M P Barr, A M Byrne, A M Duffy, C M Condron, M Devocelle, P Harriott, D J Bouchier-Hayes, J H Harmey

**Affiliations:** 1 Department of Surgery, Royal College of Surgeons in Ireland, Education & Research Centre, Beaumont Hospital, Dublin 9, Ireland; 2 Centre for Synthesis & Chemical Biology, Department of Pharmaceutical and Medicinal Chemistry, Royal College of Surgeons in Ireland, St Stephen's Green, Dublin 2, Ireland; 3 School of Biology and Biochemistry, Medical Biology Centre, Queen's University of Belfast, Northern Ireland

**Keywords:** VEGF, neuropilin-1, peptide, apoptosis

## Abstract

There is increasing evidence that vascular endothelial growth factor (VEGF) has autocrine as well as paracrine functions in tumour biology. Vascular endothelial growth factor-mediated cell survival signalling occurs via the classical tyrosine kinase receptors Flt-1, KDR/Flk-1 and the more novel neuropilin (NP) receptors, NP-1 and NP-2. A 24-mer peptide, which binds to neuropilin-1, induced apoptosis of murine and human breast carcinoma cells, whereas a peptide directed against KDR had no effect. Both anti-NP1 and anti-KDR peptides induced endothelial cell apoptosis. Confocal microscopy using 5-(6)-carboxyfluorescein-labelled peptides showed that anti-NP1 bound to both tumour and endothelial cells, whereas anti-KDR bound endothelial cells only. This study demonstrates that NP-1 plays an essential role in autocrine antiapoptotic signalling by VEGF in tumour cells and that NP1-blockade induces tumour cell and endothelial cell apoptosis. Specific peptides can therefore be used to target both autocrine (tumour cells) and paracrine (endothelial cells) signalling by VEGF.

Angiogenesis, the growth of new capillaries, is a critical process in tumour growth and metastasis (Folkman, 1995). Numerous angiogenic factors have been identified to date, the most potent of these being vascular endothelial growth factor (VEGF), otherwise known as vascular permeability factor (VPF). Anti-VEGF strategies have been shown to potently inhibit tumour growth and metastasis (Kim *et al*, 1993; Millauer *et al*, 1996; Yuan *et al*, 1996; Xu *et al*, 2000).

In endothelial cells, the biological activities of VEGF are mediated through its binding to two high-affinity tyrosine kinase receptors, Flt-1 (VEGFR-1), KDR (VEGFR-2) and its murine homologue, Flk-1 (Waltenberger *et al*, 1994). At least five isoforms of VEGF are produced as a result of alternative mRNA splicing, where these isoforms differ with regard to the expression of exons 6 and 7 of the VEGF gene. More recently, neuropilin-1 (NP-1) has been identified as an isoform-specific receptor for VEGF (Soker *et al*, 1996; Gagnon *et al*, 2000). Originally identified as a receptor for the semaphorin/collapsin family of neuronal guidance mediators (Fujisawa and Kitsukawa, 1998), NP-1 is expressed on endothelial cells and is also found on the cell surface of several tumour cells such as breast, prostate and melanoma cells. Despite a similar domain structure, NP-1 shares approximately 44% amino-acid homology with neuropilin-2 (NP-2). The NP-1-binding site in VEGF_165_ is encoded by VEGF exon 7, which is absent in the VEGF isoforms VEGF_121_ and VEGF_145_ (Soker *et al*, 1997). The related NP-2 receptor also behaves as a splice form-specific VEGF receptor that also binds VEGF_165_. Unike NP-1, NP-2 binds VEGF_145_ (Gluzman-Poltorak *et al*, 2000). Neither receptor binds VEGF_121_, which lacks the exon 7 domain.

As neuropilins lack an intracellular tyrosine kinase domain, they may act in conjunction with other cell surface receptors in order to mediate cell signalling. It has been reported that neuropilin receptors can form complexes with members of the plexin family, where the plexin is the signal transducing element of this neuropilin/plexin complex (Tamagnone *et al*, 1999; Rohm *et al*, 2000). Plexin-A1 and plexin-A2 form complexes with the neuropilins. This complex formation does not depend on the presence of semaphorin (Takahashi and Strittmatter, 2001).

Until recently, VEGF produced by tumour cells and stromal cells such as macrophages was thought to act in a paracrine manner stimulating endothelial cell growth and differentiation. However, VEGF was recently shown to be an autocrine factor for breast carcinoma invasion *in vitro* (Bachelder *et al*, 2002). We and others have also demonstrated that VEGF acts in an autocrine fashion (Bachelder *et al*, 2001; Pidgeon *et al*, 2001). Furthermore, we demonstrated that VEGF-neutralising antibodies induced apoptosis of murine 4T1 and human MDA-MB-231 mammary adenocarcinoma cells. In the present study, we examined VEGF receptor expression and the effect of peptides targeting Flk-1/KDR and NP-1 on apoptosis of 4T1 and MDA-MB-231 breast carcinoma cells.

## MATERIALS AND METHODS

### Cell culture

The murine mammary adenocarcinoma 4T1 cell line was generously provided by Dr Fred Miller (Duke University, USA). The MDA-MB-231 human mammary adenocarcinoma cell line was purchased from the American Tissue Culture Collection (ATCC). 4T1 tumour cells were maintained in RPMI 1640 medium in a humidified atmosphere of 5% CO_2_ in air at 37°C. MDA-MB-231 cells were maintained in sealed flasks with L-15 medium at 37°C. The RENCA cell line (murine renal cell carcinoma), a gift from IJ Fidler, MD Anderson Cancer Centre, USA, were grown in MEM medium supplemented with nonessential amino acids (1 ×), L-glutamine (200 mM) and sodium pyruvate (100 mM). All media were supplemented with 10% heat-inactivated fetal calf serum, penicillin (100 U ml^−1^) and streptomycin (100 *μ*g ml^−1^) (Gibco-BRL, Paisley, UK). Primary human umbilical vein endothelial cells (HUVEC), a gift from Ambrose Clarke, Department of Clinical Pharmacology, RCSI, were cultured in M-199 culture medium supplemented with 20% fetal calf serum, endothelial cell growth mitogen (Sigma, Ireland), penicillin (100 U ml^−1^) and streptomycin (100 *μ*g ml^−1^). All cells were maintained as monolayer cultures, and exponentially growing cultures were used for experiments.

### Western immunoblotting

Cells were lysed for 1 h on ice in 1 ml of lysis buffer (5 mM Tris-HCl, pH 7.4, 150 mM NaCl, 5 mM EDTA, 0.5% Triton –X-100, 0.5% SDS, 0.5% deoxycholic acid, 1 mM phenylmethylsulphonyl fluoride). Total protein concentration was determined using the Bicinchoninic Acid assay according to the manufacturer's instructions (Pierce, IL, USA).

In total, 30 *μ*g of total protein was separated on an 8% denaturing polyacrylamide gel and transferred to nitrocellulose membranes. Membranes were blocked with 5% nonfat dry milk in Tris-buffered saline (25 mM Tris-HCl, pH 7.6, 150 mM NaCl) containing 0.1% Tween-20 (TBST) for 1 h at room temperature and incubated for 60 min with NP-1 antibody (Santa Cruz Biotech, CA, USA), diluted 1 : 100 in 5% nonfat dry milk in TBST. Blots were stripped and reprobed with NP-2 antibody (Upstate Biotechnology, NY, USA), diluted 1 : 500 under similar conditions as that used for NP-1. Following six 5 min washes in TBST, membranes were incubated for 90 min with horseradish peroxidase-conjugated goat anti-rabbit antibody (DAKO, Glostrup, Denmark), diluted 1 : 2000 in TBST. Blots were probed using anti-Flt-1 and anti-Flk-1/KDR antibodies (R&D Systems, UK) at a concentration of 0.20 *μ*g ml^−1^ and using a HRP-conjugated mouse anti-goat antibody, 1 : 2000. Bound antibody was detected using enhanced chemiluminescence (Pierce, IL, USA).

### Peptide synthesis and purification

A 24-mer anti-NP1 VEGF peptide corresponding to exon 7 of VEGF_165_, CSCKNTDSRCKARQLELNERTCRC-NH_2_ (Soker *et al*, 1997), a corresponding ‘scrambled’ control peptide, NCTESKARCRLCSRCNDELTRKCQ-NH_2_, and a 7-mer anti-KDR/Flk-1 peptide, ATWLPPR-OH (Binetruy-Tournaire *et al*, 2000) were synthesised using an automated 433A peptide synthesizer (Applied Biosystems Inc., USA). Chromatographic analysis and purification were performed using a BioCAD SPRINT Perfusion Chromatography workstation (PerSeptive Biosystems) using POROS 20R2 Reversed Phase Perfusion Chromatography packing for analysis or a Jupiter Column (Phenomenex) for purification. For characterisation of peptides, Matrix Assisted Laser Desorption Ionisation-Time of Flight (MALDI-TOF) mass spectrometry was used.

### Confocal microscopy

MDA-MB-231, 4T1 and HUVEC cells were grown on glass chamber slides (Becton Dickinson, UK) and treated with 5(6)-carboxyfluorescein labelled anti-NP1 and anti-KDR peptides for 18 h. Slides were washed four times in TBS and mounted in fluorescent mounting medium (DAKO, Glostrup, Denmark). Peptide binding was examined using a Zeiss LSM 510 laser scanning confocal microscope (Carl Zeiss International, Germany). The objective used was a PLAN-NEOFLUAR 40 × /1.3 oil DIC objective.

### Flow cytometry analysis

Cells (5 × 10^4^) were seeded in six-well culture plates and treated for 24 h with VEGF control (125 *μ*g ml^−1^), anti-NP1 (125 *μ*g ml^−1^) and anti-KDR (210 *μ*g ml^−1^) peptides in medium containing 1% FCS. Cells were washed in 1 × PBS containing 2% BSA and apoptosis was assessed using the TACS™ Annexin V-FITC/PI apoptosis detection kit (R&D Systems, UK) according to the manufacturer's instructions. Flow cytometric analysis was performed using the FACSCalibur analysis system (Becton Dickinson, CA, USA). Apoptosis was measured as a percentage of the total number of cells and expressed as a percentage of controls in three independent experiments.

### Hoechst staining of apoptotic cells

Nuclear staining of apoptotic tumour cells was examined by Hoechst staining (Singhal *et al*, 1999). Cells were treated with blocking peptides, as before, for 24 h. At the end of the incubation period, cells were washed in PBS and pelleted by centrifugation at 1500 r.p.m. for 5 min. Cells (1 × 10^5^) were resuspended in 100% methanol containing Hoechst 33342 (Sigma-Aldrich, Ireland) at a final concentration of 1 *μ*g ml^−1^ and incubated at 37°C for 10 min. Cytospins were prepared, mounted in fluorescent mounting medium (DAKO, Glostrup, Denmark) and viewed by UV light microscopy (Nikon, Melville, USA).

### Statistical analysis

Statistical comparison between groups was carried out using analysis of variance (ANOVA) with LSD *post hoc* correction, using the SPSS™ statistical software package (SPSS Inc., IL, USA). Data were expressed as mean±standard error of the mean (s.e.m) and taken as significant where *P*<0.05.

## RESULTS

### Tumour cell expression of VEGF receptors

**Figure 1:**
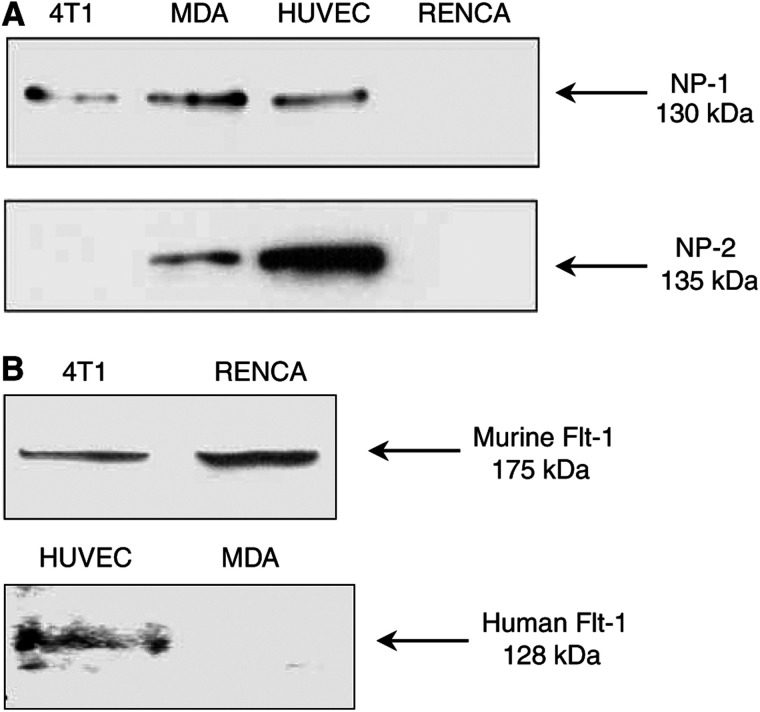
Vascular endothelial growth factor receptor expression. Tumour cells (4T1, MDA-MB-231, RENCA) and endothelial cells (HUVEC) were cultured for 24 h, and VEGF receptor expression was anlaysed by Western blot. 4T1, MDA-MB-231 and HUVEC cells expressed a 130 kDa protein corresponding to the NP-1 receptor, while MDA-MB-231 and HUVEC cells also expressed NP-2 as a 135 kDa protein (**A**). Flt-1 was identified as a 175 kDa protein in murine 4T1 and RENCA cells and as a 128 kDa protein in endothelial cells (**B**).

Vascular endothelial growth factor is constitutively expressed by murine 4T1 and human MDA-MB-231 tumour cells (4T1, 59.10±1.87 pg VEGF per*μ*g protein; MDA-MB-231, 20.56±1.51 pg VEGF per *μ*g protein, Pidgeon *et al*, 2001). In the present study, we examined the effect of blocking the binding of VEGF to the NP-1 and KDR receptors using a peptide-based approach. Western blot analysis identified NP-1 and NP-2 receptors as a 130 and 135 kDa protein, respectively, in 4T1 (NP-1), MDA-MB-231 (NP-1 and NP-2) and HUVEC (NP-1 and NP-2) cells (Figure 1A), but both were absent in the RENCA cell line (Figure 1B). Of the tumour cell lines examined, we identified the expression of Flt-1 in 4T1, RENCA and HUVEC cells only. To date, we have been unable to detect Flk-1/KDR in 4T1 and MDA-MB-231 tumour cells using immunocytochemistry and Western blotting.

### Anti-NP1 peptides bind to tumour and endothelial cells, while anti-KDR peptides bind to endothelial cells only

**Figure 2:**
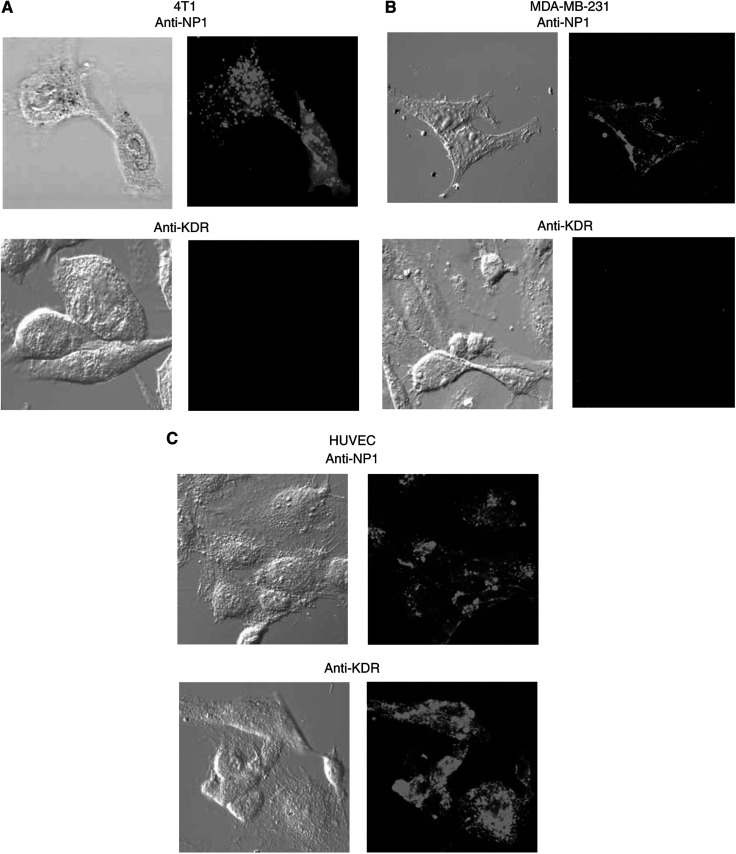
Anti-NP1 and anti-KDR peptide binding. Tumour and endothelial cells were incubated with 5-(6)-carboxyfluorescein-labelled anti-NP1 and anti-KDR peptides on chamber slides for 18 h. Peptide binding to the VEGF receptors, NP-1 and KDR, was examined on 4T1 (**A**), MDA-MB-231 (**B**) and HUVEC (**C**) cells by confocal microscopy (× 400 magnification). Images are representative of a scan zoom of between 1- and –4.2-fold.

We examined the binding of anti-NP1 and anti-KDR peptides labelled with 5(6)-carboxyfluorescein to tumour and endothelial cells by confocal microscopy. The anti-NP1 peptide bound to 4T1 (Figure 2A), MDA-MB-231 (Figure 2B) and HUVEC (Figure 2C) cells. In contrast, the anti-KDR peptide did not bind to murine 4T1 and human MDA-MB-231 tumour cells but did bind to HUVEC cells, where these are known to express functional KDR.

### Vascular endothelial growth factor receptor blockade induces apoptosis of tumour and endothelial cells

**Figure 3:**
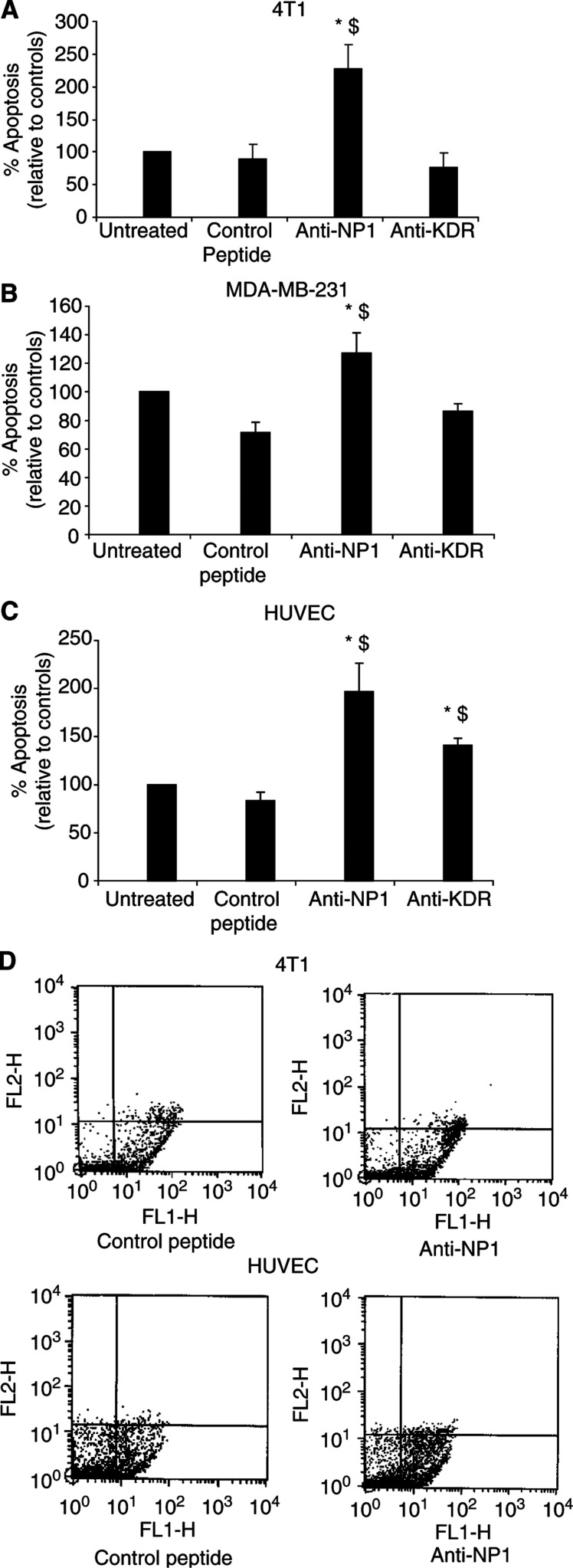
Effect of anti-NP1 and anti-KDR peptides on tumour cell and endothelial cell apoptosis. Murine 4T1 (**A**), human MDA-MB-231 (**B**) tumour cells and HUVEC endothelial cells (**C**) were treated with anti-NP1 and anti-KDR peptides or a ‘scrambled’ control peptide for 24 h. Annexin V binding to phosphatidylserine residues on the suface of apoptotic cells was assessed by flow cytometry and the percentage apoptotic cells was expressed relative to controls (mean±s.e.m) from three independent experiments (^*^P<0.05 *vs* untreated cells, ^$^*P*<0.05 *vs* control peptide). Representative dot-plots demonstrating Annexin V-positive apoptotic cells are shown in the lower right-hand quadrant following treatment of murine 4T1 tumour cells (top panel) and HUVECs (lower panel) with a scrambled control peptide and anti-NP1 peptide (**D**).

The anti-KDR peptide, ATWLPPR, was previously identified from a phage-display library and shown to block the KDR receptor, inducing apoptosis of endothelial cells (Binetruy-Tournaire *et al*, 2000). We also synthesised a 24-mer anti-NP1 peptide corresponding to exon 7 of VEGF_165_ (Soker *et al*, 1997) as the NP-1-binding motif has been shown to reside within exon 7 of this VEGF isoform. Having demonstrated that murine 4T1 and human MDA-MB-231 tumour cells express NP-1 and endothelial cells (HUVEC's) express both NP-1 and KDR, we studied the effect of anti-NP1 and anti-KDR peptides on apoptosis. HUVECs were used as a positive control as these are known to express Flt-1, KDR and NP-1 (Peters *et al*, 1993). Treatment with NP-1-blocking peptide resulted in a significant increase in apoptosis of both tumour cells and endothelial cells (4T1, 226.72±38.80%; MDA-MB-231, 126.99±14.49%; HUVECs, 196.77±29.51%, *P*<0.05) relative to cells treated with a scrambled control peptide (4T1, 88.66±22.77%; MDA-MB-231, 71.45±7.32%; HUVECs, 83.40±8.78%, p<0.05, Figure 3A, B and C, respectively). Anti-KDR peptide induced significant apoptosis of HUVECs relative to controls (141±7% *vs* control, *P*<0.05) but had no effect on tumour cell apoptosis in murine and human tumour cells (4T1, 77.12±20.82%; MDA-MB-231, 86.24±5.61% *vs* control), consistent with the lack of expression of Flk-1/KDR in these cells. Representative dot plots showing induction of tumour cell (4T1) and endothelial cell (HUVEC) apoptosis by anti-NP1-blocking peptide relative to a VEGF control peptide are shown (Figure 3D), following analysis by flow cytometry. RENCA cells were also treated with anti-NP1 and anti-KDR-blocking peptides as previously described. These cells do not express the VEGF receptors NP-1 or Flk-1. As expected, neither peptide induced apoptosis of these tumour cells (data not shown), demonstrating that the anti-NP1 and anti-KDR peptides only induce apoptosis in NP-1-positive or KDR-positive cells, respectively.

To demonstrate that the induction of apoptosis in NP1-expressing 4T1 and MDA cells by anti-NP1 peptide was not a consequence of nonspecific cytotoxicity, RENCA cells, which do not express the NP1 receptor, were treated with a scrambled control peptide and the anti-NP1 peptide. As expected, neither peptide induced apoptosis in NP1-negative RENCA cells.

### Morphological evaluation of tumour cell apoptosis using anti-NP1 and anti-KDR blocking peptides

**Figure 4:**
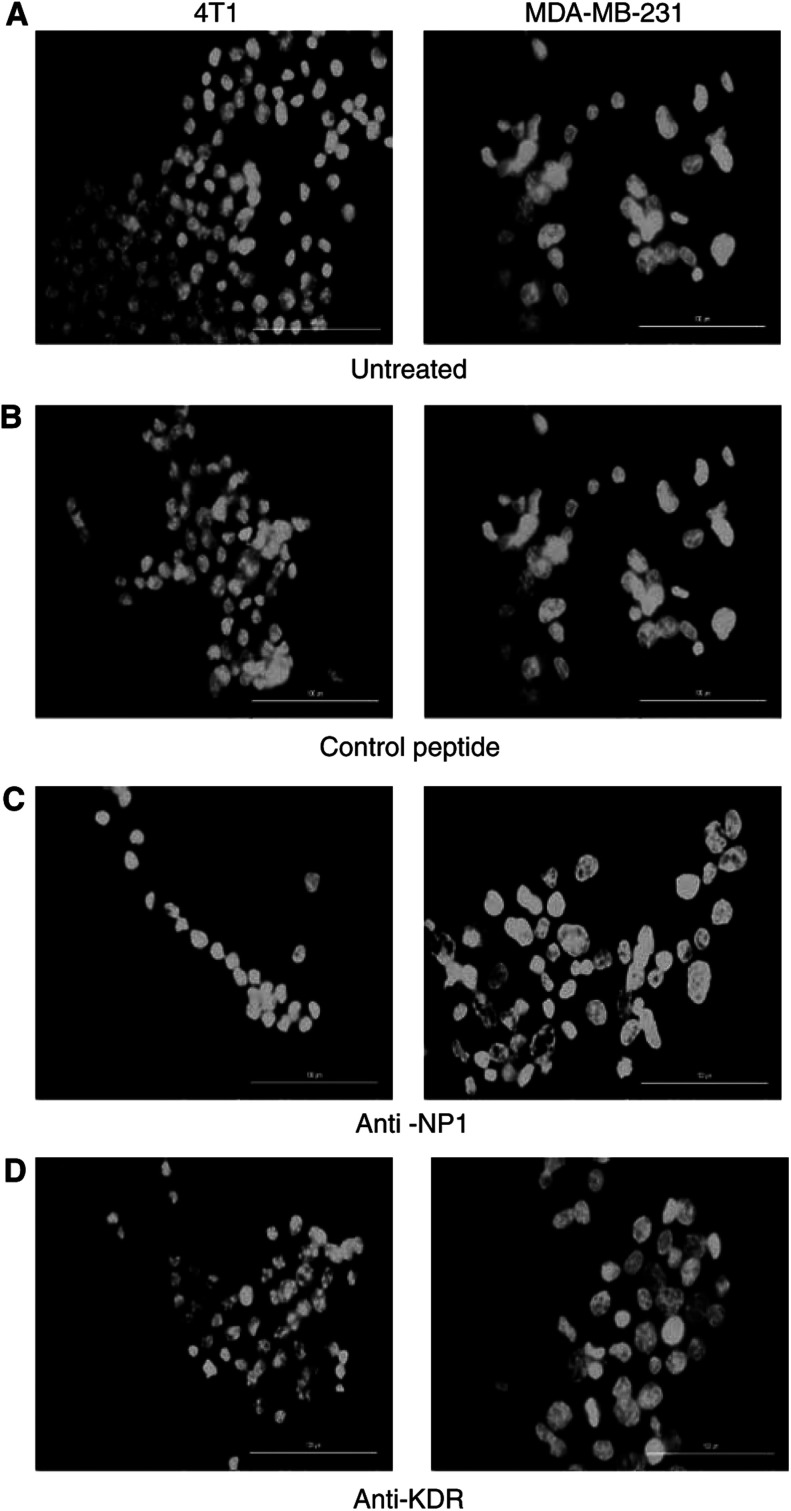
Hoechst staining of apoptotic tumour cells. 4T1 and MDA-MB-231 tumour cells were incubated for 24 h with control peptide (**B**), anti-NP1 (**C**) and anti-KDR (**D**) peptides. Following incubation, cells were stained with Hoechst 33342 (1 *μ*g ml^−1^) in methanol and examined under UV light (× 400 magnification). Treatment of 4T1 and MDA-MB-231 tumour cells with anti-NP1 peptides induced apoptosis, as seen by bright fluorescing cells relative to control peptide and cells treated with anti-KDR peptide.

To confirm the induction of 4T1 and MDA-MB-231 tumour cell apoptosis using anti-NP1 blocking peptides, nuclear morphology was examined by Hoechst 33342 staining. Tumour cells treated for 24 h with anti-NP1 blocking peptide (Figure 4C) demonstrated typical apoptotic nuclei relative to untreated cells (Figure 4A) or cells treated with a scrambled control peptide (Figure 4B). Anti-KDR peptides however did not induce apoptosis of 4T1 and MDA-MB-231 tumour cells (Figure 4D). These findings at the morphological level are consistent with our findings using Annexin V-FITC/PI by flow cytometry.

## DISCUSSION

In contrast to the classical VEGF receptors, Flt-1 (VEGFR-1) and Flk-1/KDR (VEGFR-2), and the mechanisms of VEGF signalling via these tyrosine kinase receptors, relatively little is known regarding the mechanisms governing VEGF signalling via the neuropilins.

A review of the literature of VEGF receptors and MDA-MB-231 tumour cells appears controversial, with a number of groups reporting different VEGF receptor profiles. Soker *et al* (1998) report that NP-1 is the only VEGF receptor associated with these cells. This is consistent with the findings from Bachelder *et al* (2001) who also failed to identify KDR expression by Western blot. However, Tae-Hee *et al* (2003) found detectable levels of Flk-1/KDR mRNA in MDA-MB-231 cells by Northern blot analysis. Such findings are consistent with our observations using RT–PCR. Despite a lack of evidence for Flt-1 expression at the protein level, we detected Flt-1 mRNA (data not shown). This is in contrast to that reported by Soker *et al* (1996) where they report undetectable levels of Flt-1 mRNA. Similarly, there have been a number of contrasting reports regarding NP-2 expression by MDA-MB-231 cells. Using ^125^I-VEGF_145_- and ^125^I-VEGF_165_-binding studies, Gluzman-Poltorak *et al* (2000) deduced that MDA-MB-231 tumour cells do not express detectable levels of functional NP-2 receptors. Mamluk *et al* (2002) on the other hand report NP-2 expression on these cells. Our findings that MDA-MB-231 cells and endothelial cells express NP-2 at the protein level are in agreement with those reported by the latter. We found that 4T1 tumour cells express only NP-1 and Flt-1 receptors.

It has recently been reported that MDA-MB-231 breast carcinoma cells express sema-3A and plexin-A1, which can both bind to NP-1. Indeed Sema-3A and VEGF compete for binding to NP-1. These act as antagonistic autocrine NP-1 ligands that regulate breast carcinoma cell migration (Bachelder *et al*, 2003). Plexin-A1 and plexin-A2 can form complexes with NP-1 and NP-2. Having found NP-1 and NP-2 as the only VEGF receptors expressed by these cells, we propose that VEGF signalling in MDA-MB-231 cells may be mediated by plexin/NP complexes. Since Bachelder *et al* demonstrated a role for such receptors in tumour cell chemotaxis, these receptors may also play a functional role in VEGF-mediated tumour cell survival. Since 4T1 tumour cells only express NP-1 and Flt-1, both receptors may interact with each other in order to transduce signalling via the classical tyrosine kinase receptor, Flt-1. Alternatively, such signalling may be mediated via plexin-A1. In endothelial cells (HUVEC), there exists a number of possibilities whereby VEGF mediates signalling through a number of its receptors. Evidence to date suggests that VEGF signalling in endothelial cells occurs via the formation of complexes involving KDR and NP-1. This in turn enhances the binding of VEGF_165_ to KDR. Both KDR and NP-1 play an important role in VEGF-mediated survival signalling as blockade of either receptor induces apoptosis of endothelial cells.

In conclusion, we have demonstrated that a 24-mer peptide targeting the VEGF_165_-binding site on the novel VEGF receptor, NP-1, antagonises the autocrine antiapoptotic effects of VEGF on breast carcinoma cells. The use of peptides over monoclonal antibodies offer a number of advantages including low manufacturing costs, lower risk of an immune response, improved organ and tumour penetration and greater stability. Owing to their reduced size, peptides offer an attractive therapeutic option over antibodies as inducers of apoptosis and angiogenesis inhibitors. As chemo/radiotherapy increases VEGF expression (Gorski *et al*, 1999), an autocrine role for VEGF in protecting tumour cells from apoptosis may explain the synergistic effect of VEGF blockade when combined with chemo/radiotherapy (Harmey and Bouchier-Hayes, 2002). Thus, it is likely that anti-NP1 peptides will be most effective when used in combination with other antiangiogenic strategies and traditional apoptosis-inducing chemotherapy/radiotherapy regimens.
